# 

**Published:** 1837-04

**Authors:** 


### CONTENTS OF No. VI.

OF THE

### British and Foreign Medical Review.

APRIL, 1837.

---

### Part First.—Analytical and Critical Reviews.

PAGE

Art. I.—1. A Further Inquiry concerning Constitutional Irritation, &c. By Benjamin Travers, f.r.s. . . . . . 301
2. Outlines of Human Pathology. By Herbert Mayo, f.r.s. . . ib.
3. An Essay on Laryngismus Stridulus. By Hugh Ley, m.d. . . ib.
4. Researches on the Diseases of the Brain. By John Abercrombie, m.d. . ib.
5. Lectures on the Nervous System. By Marshall Hall, m.d. . . ib.
6. Papers on Diseases of the Brain, (Med.-Chir. Transac. vol. xix.) By J. Sims, m.d. ib.
7. Isis Revelata. By J. C. Colquhoun, Esq. . . . . . ib.

Art. II.—1. Clinical Illustrations of the more important Diseases of Bengal, with the Result of an Inquiry into their Pathology and Treatment. By William Twining, First Assistant Surgeon, General Hospital, Calcutta. 2d Edition. 329
2. A Treatise on the Functional and Structural Changes of the Liver in the Progress of Disease; and on the Agency of Hepatic Derangement in producing other Disorders; with numerous Cases exhibiting the Invasion, Symptoms, Progress, and Treatment of Hepatic Disease in India. By W. E. E. Conwell, Surgeon of the Madras Establishment . . . . . ib.
3. Transactions of the Medical and Physical Society of Calcutta. Vols. VI. and VII. ib.
4. The Indian Journal of Medical Science. Edited by F. Corbyn, Esq. Vol. III. ib.

Art. III.—Practical Observations on Strangulated Hernia, and some of the Diseases of the Urinary Organs. By Joseph Parrish, m.d. . . . 355

Art. IV.—Researches into the Physical History of Mankind. By James Cowles Prichard, m.d. f.r.s. &c. . . . . . . 365

Art. V.—An Essay on the Origin and Nature of Tuberculous and Cancerous Diseases. By Richard Carmichael, m.r.i.a. &c. . . . . 376

Art. VI.—Essai sur la Philosophie Médicale, et sur les Généralités de la Clinique Médicale; precédé d'un Resumé Philosophique des principaux Progrès de la Médecine, &c. Par J. Bouillaud, Professeur de Clinique Médicale, &c. 384
An Essay on Medical Philosophy, and on the Generalities of Clinical Medicine, &c. Par J. Bouillaud, &c.

Art. VII.—The Principles and Practice of Obstetric Medicine, in a Series of Systematic Dissertations on Midwifery and on the Diseases of Women and Children. Illustrated by numerous Plates. By David D. Davis, m.d. &c. 392
2. Operationslehre für Geburtshelfer, in zwei Theilen. Von Dr. Hermann Friedrich Kilian, ordentl. öffentl. Professor der Geburtshülfe und der Geburtshülflichen Klinik an der Rheinischen Friedrich-Wilhelms Universität, u. s. w. Erster Theil: die Operative Geburtshülfe . . . 411
Instructions in Operating for Accoucheurs, in two Parts. By Hermann F. Kilian, m.d. &c. Part First: Operative Midwifery.

Art. VIII.—The Nature and Treatment of Dropsy; considered especially in reference to the Diseases of the internal Organs of the Body which most commonly produce it. Parts I. and II. Anasarca and Ascites. With an Appendix, containing a Translation of the Work of Dr. Geromini on Dropsy, from the original Italian. By Edward J. Seymour, m.d., &c. . . 429

Art. IX.—A Practical Treatise on the Management and Diseases of Children. By Richard T. Evanson, m.d., &c., and Henry Maunsell, m.d. &c. . 439

Art. X.—Durstellungen und Ansichten zur Vergleichung der Medicin in Frankreich, England, und Deutschland, nach einer Reise in diesen Ländern im Jahre 1835. Von Dr. Adolph Mühry, Practischem Arzte und Wundarzte, &c. . 447

Comparative View of the State of Medicine in France, England, and Germany, from a Tour made in those Countries during the Year 1835. By Dr. Mühry.

PAGE

ART. XI.—1. Elements of the Practice of Physic, presenting a View of the present State of Special Pathology and Therapeutics. By DAVID CRAIGIE, M.D. &c. 452
2. Elements of the Practice of Medicine. By RICHARD BRIGHT, M.D., and THOMAS ADDISON, M.D. &c. . . . . . . . ib.
3. Elements of Medicine. Vol. I. On Morbid Poisons. By ROBT. WILLIAMS, M.D. ib.

ART. XII.—Researches on the Effects of Bloodletting in some Inflammatory Diseases, and on the Influence of Tartarized Antimony and Vesication in Pneumonitis. By P. C. A. LOUIS, M.D. &c. Translated by C. G. PUTNAM, M.D.; with Preface and Appendix, by JAMES JACKSON, M.D. &c. . 456

ART. XIII.— THE FOREIGN JOURNALS. No. VI.

GERMAN JOURNALS:

12. Hannoversche Annalen für die gesammte Heilkunde. Eine Zeitschrift. Herausgegeben von Dr. G. P. HOLSCHER, &c. . . 460
Hanoverian Annals of the Medical Sciences. Edited by Dr. HOLSCHER.

13. Wochenschrift für die gesammte Heilkunde. Herausgeber, Dr. CASPER, 461
Weekly Journal of Medical Science. Editor, Dr. CASPER, &c.

14. Jehrbücher der In- und Ausländischen Gesammten Medecin. Herausgegeben von C. C. SCHMIDT, M.D. . . . . . 462
Annals of Medical Science, Domestic and Foreign.

---

### PART SECOND.—Bibliographical Notices.

ART. I.—A Treatise on Tetanus; being the Essay for which the Jacksonian Prize, for the year 1834, was awarded by the Royal College of Surgeons, London. By THOMAS BLIZARD CURLING, Assistant Surgeon to the London Hospital 463

ART. II.—Supplemente zur Lehre vom Kreislaufe. II. Heft. Flimmerbewegungen, Leben der Blutsphären, Monadenlehre. Von Dr. A. F. I. C. MAYER, &c. 465

Supplement to the Doctrine of the Circulation. Second Part. Ciliary Motions, Life of Blood-globules, Doctrine of Monads. By Dr. MAYER.

ART. III.—The Fallacy of the Art of Physic as at present taught in the Schools, with the Development of new and important Principles of Practice. By SAMUEL DICKSON, M.D. . . . . . . . 469

ART. IV.—Thoughts on Physical Education, and the true Method of Improving the Condition of Man. By CHARLES CALDWELL, M.D.; with Notes, by ROBERT COX; and a recommendatory Preface, by GEORGE COMBE . . 472

ART. V.—Rocznik obejmujancy zdanie sprawy z czynnos'ci Kliniki Chirurgicznéj Uniwersytetu Jagiellon'skiego, od 1go Paz'dziernika, 1833, do 1go Lipca, 1834, roku. Wydal LUDWIK BIERKOWSKI, Dyrektor rzeczonego Zakladu, &c. . 475

Annual Report of the Surgical Clinique of the Jagellonian University, from the 1st of October, 1833, to the 1st of July, 1834. Published by L. BIERKOWSKI.

ART. VI.—1. Catalogue Raisonné; or, Classified Arrangement of the Books in the Library of the Medical Society of Edinburgh . . . . 476

2. An Address delivered to the Members of the Royal Medical Society (of Edinburgh), December 16th, 1836. By JOHN H. BENNETT, President of the Society . 477

ART. VII.—An Elementary System of Physiology. By JOHN BOSTOCK, M.D. &c. ib.

ART. VIII.—Observations on the Medical and Surgical Agency of the Air-Pump. By Sir JAMES MURRAY, M.D. . . . . . . 478

ART. IX.—The Principles of Surgery. By JAMES SYME, F.R.S.E. &c. . . 481

ART. X.—1. Mittheilungen ueber die Cholera Epidemie zu St. Petersburg, im Sommer 1831. Unter redaktion der Herrn D. LICHTENSTADT und SEIDLITZ 484

Communications respecting the Epidemic Cholera at St. Petersburg, &c.

2. Rapport sur le Cholera Morbus de Moscou. Par F. C. MARCUS, M.D. &c. . ib.

Report on the Cholera Morbus of Moscow, &c.

3. Specimen Historico-Medicum de Choleræ Asiaticæ itinere per Belgium Septentrionale. Auctore A. C. G. SUERMAN . . . . . ib.

A Medico-Statistical Account of the Epidemic Cholera in Holland, &c.

ART. XI.—The Works of JOHN HUNTER, F.R.S.; with Notes. Edited by JAMES F. PALMER, Senior Surgeon to the St. George's and St. James's Dispensary . 486

ART. XII.—Traité de la Consomption Pulmonaire, &c. Par JAMES CLARK, M.D. Traduit de l'Anglais, par HENRI LEBEAU, &c. . . . . 487

A Treatise on Pulmonary Consumption, &c. By JAMES CLARK, M.D. &c.

ART. XIII.—Illustrations of the Comparative Anatomy of the Nervous System. By JOSEPH SWAN. Part II. . . . . . . 489

PAGE

ART. XIV.—A Description of the Bones, together with their several Connexions with each other and with the Muscles; especially adapted for Students in Anatomy. By JOHN F. SOUTH, Assist. Surgeon to St. Thomas's Hospital . 491

ART. XV.—Sketch of the Comparative Anatomy of the Nervous System; with Remarks on its Development in the Human Embryo. By JOHN ANDERSON 491

ART. XVI.—Outlines of a Course of Lectures on Medical Jurisprudence. By Dr. TRAILL 492

ART. XVII.—Observations upon the Construction and Use of the Respirator, &c. By JULIUS JEFFREYS, Surgeon . . . . . ib.

ART. XVIII.—An Essay on the Influence of Air and Soil as affecting Health. By ALEXANDER WRIGHT, Student . . . , . 494

ART. XIX.—Memoranda for Young Practitioners in Midwifery. By EDWARD RIGBY, M.D., Lecturer on Midwifery at St. Thomas's Hospital, &c. . ib.

### PART THIRD.—Selections from Foreign Journals.

### ANATOMY, PHYSIOLOGY, AND PATHOLOGY.

On Internal Strangulations of the Intestines. By Dr. CHARLES ROKITANSKY . 495
On the Ciliary Motions in the Brain. By M. PURKINGE . . . 501
On the Ciliary Motions in Man. By C. T. VON SIEBOLD . . . 502
On Secondary Abscess. By Dr. H. NASSE, of Bonn . . . ib.
Observations on the best Mode of Demonstrating the Internal Structure of the Heart, and on the Septum of the Auricles in Man. By Professor RETZIUS 506

### PRACTICAL MEDICINE AND THERAPEUTICS.

Remarkable Case of Spontaneous Combustion. By Dr. JOLY . . 507
Employment of the Root of the Elder in Scrofula and Leucorrhœa. By M. DELENS 508
Employment of Creosote and Tar-water in Pulmonary Affections. By M. PETREQUIN 509
Chronic Fluor Albus, cured by Iodine. By Dr. MÜLLER . . . ib.
Influence of certain Medicines upon the Functions of the Heart. By M. LOMBARD 510
Iodine in Mercurial Salivation. By M. KLOSE . . . . . 511
On the Diuretic Operation of the Flowers of Statice Armeria. By Dr. EBERS . ib.
Intermittent Epistaxis, cured by Quinine . . . . ib.
Cure of Hydrops Articuli, on the occurrence of Intermittent Fever . . ib.

### SURGERY.

On the Treatment of Vesico-Vaginal Fistulæ. By Professor DIEFFENBACH . ib.
On Pessaries, and Radical Cure of Prolapsus Vaginæ et Uteri. By Pr. DIEFFENBACH 512
Treatment of some Forms of Acute Ophthalmia by Blisters. By A. VELPEAU . 513
Treatment of Club-foot with Plaster of Paris. By Professor DIEFFENBACH . 514
On the Permanent Immoveable Bandage. By Dr. SUETIN . . . 515
Local Treatment of certain Forms of Venereal Disease in Berlin. By Dr. STRUNZ 516
Cure, after Excision of a Portion of Intestine. By Professor DIEFFENBACH . 517
Report of the Cases treated at the Ophthalmic Hospital at Vienna. By Prof. ROSAS ib.
Treatment of Artificial Anus. By M. BLANDIN . . . . 520
Treatment of Chronic Catarrh of the Bladder, by Injections. By M. DIVERGIE, sen. 521
Dissection of a Sclerotic Staphyloma. By Dr. JAEGER, of Erlangen . . ib.
Case of Ligature of the Common Carotid. By Dr. BEDOR . . . 522

### MIDWIFERY.

Case of Extra-Uterine Pregnancy. By WILLIAM BELL, M.D. . . ib.
On the Reposition of the Umbilical Cord. By Dr. MICHAELIS . . 523
Case of Delivery after Episioraphy. By Dr. PLATT, of Hamburg . . 524
On the Use of the Plug in Cases of Placenta Prævia. By Dr. ALBERT . . 525
On the Means of inducing Uterine Contraction. By Dr. SCHNEIDER . . ib.
Contributions to the Pathology of Puerperal Fever. By Dr. A. BARTELS . ib.

### MEDICAL STATISTICS.

Prevalence of Consumption in the United States of America. By Dr. BRIGHAM . 526

### HYGIENE.

Poisoning by Arsenic mixed with Beans. By MM. BARRUEL and CHEVALLIER . 528

### ANIMAL CHEMISTRY.

On the Distinctive Characters of Pus, &c. By Dr. A. DONNÉ . . 530

### FORENSIC MEDICINE.

Case of Wounds produced by Sulphuric Acid. Feigned Insanity. By M. BOPP . 533
Case of Death from the Rupture of an Intercostal Artery, &c. By Dr. GRAFF . 536

### TOXICOLOGY.

Case of Poisoning by Sulphuric Acid. Death after twelve Weeks. By Dr. BRAUN 539

### Part Fourth.—Selections from British Journals.

### ANATOMY, PHYSIOLOGY, AND PATHOLOGY.

PAGE
On the Motions and Sounds of the Heart. By Drs. Williams, Todd, &c. . 541
On the Chemistry of the Digestive Organs. By R. D. Thomson, m.d. . ib.
Physical Signs of the Height of the Diaphragm in the Chest. By E. Harrison, m.d. 542
On the Causes of the Resonance in Percussion. By John Clendinning, m.d. . ib.
Theory and Practice of Percussion as a Mode of Diagnosis. By Dr. Williams . 543
On the Tubulo-fibrous Structure of the Brain. By Andrew Pritchard, Esq. . 547
On the Cause of the Second Sound of the Heart. By Thomas Watson, m.d. . ib.

### PRACTICAL MEDICINE AND THERAPEUTICS.

On Dropsy. By Dr. Mateer, of Belfast . . . . 548
A Letter on Typhus Fever. By Robert Perry, m.d., of Glasgow . . ib.
Case of Ulceration of the Brain. By A. J. Hannay, Professor of Physic . 549
Observations on Cerebral and Spinal Apoplexy, Paralysis, and Convulsions of New-born Infants. By Evory Kennedy, m.d. . . . 550
Researches on the Symptoms and Diagnosis of Aneurismal and other Tumours in the Cavity of the Thorax. By George Greene, m.d . . 551
Poisonous Effects of Black Oxide of Manganese. By John Cowper, m.d. . 552
Third Report of Medical Cases treated at St. George's Hospital. By Dr. Macleod ib.
On Pectoriloquy. By Edwin Harrison, m.d. . . . . 554
Remarks on the Nature and Treatment of Rheumatism. By J. Hope, m.d. . 555
Case of Perforation of the Stomach. By J. C. Parkin, Surgeon . . 556

### SURGERY.

On the Nature and Treatment of Lupus. By Alexander Ure, m.d. . . ib.
New Mode of Operating for Nævus. By Mr. Liston . . . 557
Cure of an Open Cancer of the Breast. By Andrew Buchanan, m.d. . ib.
Gangrene of the Leg from Disease of the Heart. By W. Clark, Esq. . ib.
On the Treatment of Erysipelas by Bandaging. By James Allan, Esq. . 558
Report of Cases treated in the Glasgow Royal Infirmary. By J. Macfarlane, m.d. ib.
On the Treatment of Hydrocele by Acupuncture. By D. Lewis . . 559
Account of a Method of Operating for Hydrocele. By Benjamin Travers, Esq. 560
On Hydriodate of Potass in Secondary Syphilis. By H. Bullock, m.r.c.s. . 561
Extirpation of an Inverted Uterus. By W. Moss, Esq. . . . ib.
Unusual State of the Contents of the Hernial Sac. By Henry Taynton, Esq. . 562
On Union of Fractures of the Patella. By George Gulliver, Assistant Surgeon ib.
Case of Scalded Glottis. By George Fayrer, Surgeon . . . ib.

### MIDWIFERY.

Two Cases of complete Recovery from Laceration of the Posterior Part of the Vagina and Cervix Uteri. By J. Toogood, Esq. . . . ib.

### MEDICAL STATISTICS.

1. On a Method of determining the Danger and the mean future Duration of Diseases at different Periods of their Progress. 2. On the Law of Recovery and Dying in Small-Pox. By William Farr, Esq. . . . 563

### MEDICAL JURISPRUDENCE.

On the Morbid Appearances in Death by Drowning. By W. Ogston, m.d. . 564

### TOXICOLOGY.

On the Poisonous Effects of the Berries of the Yew. By S. Hurt, Esq. . ib.

---

### Part Fifth.—Medical Intelligence.

On the Present State of Medicine in Denmark, (Second Report) . . 565
State of Medicine in America . . . . . 575
On the Present State of Medicine in Italy . . . . 576
Dr. M. Hall's Discovery in reference to the Functions of the Spinal Marrow . 577
Scientific Discoveries in Germany . . . . . 582
Royal Medical Society of Edinburgh . . . . . 583
OBITUARY—Professor Turner . . . . . 585
John Johnstone, m.d. f.r.s. Hugh Ley, m.d. Dr. Macnish . 586
Books received for Review . . . . . 587

